# The role of affective temperaments on depressive rumination in individuals with bruxism

**DOI:** 10.3934/publichealth.2026002

**Published:** 2026-01-05

**Authors:** Clara Lombardo, Cosimo Galletti, Gabriele Cervino, Chiara La Barbiera, Clemente Cedro, Gianluca Pandolfo, Maria Rosaria Anna Muscatello, Enrico Nastro Siniscalchi, Mario Nicolas De La Fuente, Sofia Munge, Sergio Parra García, Giuseppe Alberti, Daniele Portelli, Carmela Mento

**Affiliations:** 1 Department “Scienze della Salute”, University of Catanzaro, Catanzaro, Italy; 2 Faculty of Medicine and Surgery, Kore University of Enna, Enna, Italy; 3 Department of Dental Research Cell, Dr. D. Y. Patil Dental College & Hospital, Dr. D. Y. Patil Vidyapeeth (Deemed to be University), Pimpri, Pune, India; 4 Department of Biomedical and Dental Sciences and Morphofunctional Imaging, University of Messina, Messina, Italy; 5 Departamento de Medicina Córdoba, Universidad Católica de Córdoba, Argentina; 6 Department of Surgery, University of Salamanca, Salamanca, Spain; 7 Unit of Otorhinolaryngology, Department of Adult and Development Age Human Pathology “Gaetano Barresi”, University of Messina, Messina, Italy

**Keywords:** bruxism, affective temperaments, depressive rumination, oral health, mental health

## Abstract

Bruxism, defined as the grinding or clenching of teeth, is a multifactorial condition whose etiology involves both psychological and psychopathological aspects. In particular, it has been associated with variables such as anxiety, depression, and ruminative thinking. The aim of the present study is to examine the psychological characteristics associated with bruxism, with a particular focus on affective temperaments and depressive rumination. The subjects recruited from the general population were assessed through an online survey including the Ruminative Response Scale (RRS) and the Temperament Evaluation of Memphis, Pisa, Paris, and San Diego Autoquestionnaire (TEMPS-A). Group differences were analyzed using the student's t-test for independent samples. Furthermore, a linear regression analysis was performed, thereby considering the RRS as the dependent variable and the TEMPS-A temperament dimensions as independent variables, in order to identify which temperamental profiles could explain depressive rumination in individuals with bruxism. The analyses revealed significant gender differences in the cyclothymic and anxious temperaments, as well as in the “Brooding” and “Depression” dimensions of the RRS. A regression analysis further indicated that cyclothymic and depressive temperaments predicted “Brooding” and “Depression”, whereas only the cyclothymic temperament emerged as a predictor of “Reflection”. These findings highlight the relevance of affective temperaments and depressive rumination in the psychological profile of patients with bruxism, thus underlining their importance for clinical practice.

## 1. Introduction

Bruxism is a repetitive activity that involves the jaw muscles, characterized by the clenching or grinding of teeth, as well as mandibular bracing or thrusting. This condition can occur both during night-time rest and while awake [1],[2]. Bruxism can be classified as primary, when it arises without any underlying medical condition, or secondary, when it is associated with psychiatric or medical disorders [3]. Recent epidemiological research estimates that bruxism affects approximately 22.22% of the global population, with sleep bruxism occurring in 21% of individuals and awake bruxism in 23% [4]. Due to its high prevalence and strong links with stress, bruxism is increasingly recognized as a condition that affects a person's psychological well-being and quality of life. Psychological factors, including emotional traits and cognitive styles, may contribute to the development and maintenance of bruxism. Understanding the psychological correlations of bruxism require examining affective temperaments, the stable emotional dispositions linked to behavior, and mood regulation. According to Akiskal's framework, different affective temperaments are associated with distinct emotional and behavioral tendencies [5]. The depressive temperament is characterized by persistent low mood, introversion, reduced energy levels, and a heightened need for sleep. In contrast, the hyperthymic temperament is marked by an expansive mood, high activity levels, and minimal sleep requirements. Individuals with a cyclothymic temperament disposition have frequent and unpredictable shifts in mood, energy, and sleep-wake patterns, while those with an irritable temperament exhibit a pronounced tendency toward aggression and confrontational behaviors. Finally, the anxious temperament is associated with heightened vigilance, insecurity, somatic distress, and difficulty in relaxation. These temperaments, assessed with the Brief Italian Version of the Temperament Evaluation of Memphis, Pisa, Paris, and San Diego Autoquestionnaire (TEMPS-A), remain relatively stable across the lifespan and contribute to personality development [6]. Psychological factors, including anxiety, depression, and ruminative thinking, have been widely recognized as key contributors to the etiology of bruxism [7]–[9]. Dental anxiety and stress related to anticipated dental treatments may increase muscle tension, thus promoting teeth clenching or grinding and exacerbating bruxism-related behaviors [10],[11]. Depressive rumination is a cognitive process that involves repetitive, passive thoughts about depressive symptoms, their causes, and the possible consequences. According to Nolen-Hoeksema's Response Styles Theory, this pattern of thinking not only accompanies depressive states but also contributes to their persistence, thus preventing the adoption of more adaptive coping strategies [12]. In this context, rumination represents a common response to negative emotional states, thereby fostering a cycle of repetitive thoughts that intensify psychological distress. This phenomenon can amplify physiological stress responses and contribute to the persistence of bruxism-related behaviors [13],[14]. Other conditions, such as self-perceived halitosis, have also been linked to negative psychological effects such as anxiety and social discomfort. These factors may impair emotional regulation and promote ruminative thinking, as observed in individuals with bruxism [15]. This study uses the Ruminative Response Scale (RSS), a widely used instrument to assess depressive rumination, consisting of the following sub-scales: Brooding, which focuses on self-critical and negative thoughts; Reflection, which involves more contemplative but maladaptive processing of distressing thoughts; and Depression, which captures thoughts related to depressive symptoms and their causes [16]. Recent studies have highlighted the role of rumination within affective temperaments, thereby indicating that brooding, in particular, is strongly associated with heightened emotional distress and may exacerbate psychological symptoms [17]. This analysis provides further insight into how affective temperaments may influence depressive rumination in patients with bruxism, thereby identifying the temperaments that most significantly predict this behavior. In light of this, the aim of the present study is to examine the psychological characteristics associated with bruxism, with a particular focus on affective temperaments and depressive rumination.

## 2. Materials and methods

The data were recorded using an anonymous, publicly accessible online survey via Google Forms (Google LLC, Mountain View, CA, USA) that was disseminated through social (websites, Facebook, and Twitter) from March to December 2024. Only complete forms were retained and examined. The study complied with the principles of the Declaration of Helsinki and only involved healthy participants. All data were fully anonymized, and informed consent was obtained at the time of the original data collection; therefore, additional ethical approval was not required. The inclusion criteria were age ≥ 18 years and self-reported symptoms of bruxism. The presence of bruxism was determined through self-assessment, using screening questions. The participants were classified as having bruxism if they reported at least one of the following behaviors: clenching or grinding their teeth during sleep, clenching their jaw while awake, or tension or fatigue in their maxillary muscles upon waking. The exclusion criteria included the following: history of neurological or psychiatric disorders, use of drugs acting on the central nervous system, or incomplete responses to psychological questionnaires.

### 2.1. Measures

In order to achieve our goals, the following psychological tests were administered:

- TEMPS-A is a validated instrument for assessing affective temperaments. The Italian short version was deemed more appropriate for large-scale studies as compared to the original 110-item version. This abbreviated form consists of 39 dichotomous (Yes/No) items, which allows for the evaluation of five affective temperament subscales: cyclothymic, depressive, irritable, hyperthymic, and anxious. These temperaments are considered subclinical traits that may increase the susceptibility to specific mood disorders. The reliability of all TEMPS-A subscales, assessed using Cronbach's *α*, demonstrated a satisfactory internal consistency (*α* > 0.70) [6],[18].

- RRS is a self-report instrument widely used to assess the tendency to engage in rumination, which is a maladaptive cognitive process characterized by repetitive and passive attention to one's distress and its causes and consequences. The RRS, originally developed by Nolen-Hoeksema and Morrow (1991) and later revised by Treynor et al. (2003), consists of 22 items rated on a Likert scale ranging from 1 (almost never) to 4 (almost always). The scale was organized into 3 dimensions: Brooding, Reflection, and Depression. The RRS is considered a reliable and valid measure of ruminative tendencies, with a good internal consistency (Cronbach's *α* typically > 0.70) and significant associations with depressive symptoms and other affective disorders. Given its robust psychometric properties, the RRS is widely used in clinical and research settings to investigate the role of rumination in emotional regulation and psychopathology [19],[20].

### 2.2. Statistical analysis

Continuous data were reported as mean ± standard deviation, while internal reliability was separately verified for the two scales and for each dimension by calculating Cronbach's alpha [21]. Differences between the groups were analysed using the Student's *t*-test for independent samples. Subsequently, a linear regression analysis was conducted, in which the RRS variables (Brooding, Reflection, Depression) were considered as dependent variables and all TEMPS-A factors were included as predictors to identify which temperamental dimensions could serve as specific predictors of depressive rumination in subjects with bruxism. A Bonferroni correction was applied to adjust for multiple comparisons. Since eight independent group comparisons were conducted (three RRS dimensions and five TEMPS-A temperament factors), the corrected significance threshold was calculated as 0.05/8 = 0.006. After applying the Bonferroni correction, the Cyclothymic and Depressive temperaments remained significant predictors of the dimensions “Rumination” and “Depression”, while only the Cyclothymic temperament retained significance for the dimension “Reflection”. These results confirm the strength of the associations between affective temperaments and the dimensions of depressive rumination. Results with *p* ≤ 0.006 were considered statistically significant. A statistical analysis was performed using the Statistical Package for the Social Sciences (SPSS) version 25.0 (SPSS Inc., Chicago, IL, USA).

## 3. Results

Of 581 eligible subjects, 338 were excluded because they did not present bruxism symptoms. The final sample of 243 participants consisted of 57 men and 186 women, with an average age of 31.58 (*S.D*. 9.18). The data indicated that 74% of the subjects were college graduates and 41.3% were employed. Among the study participants, 66.1% reported experiencing sleep disturbances, while 51.2% suffered from night terrors. Additionally, 52.4% woke up with muscle tension in the mouth and jaw, particularly in the morning. During the day, 65.7% unconsciously clenched their jaw and 52.4% noticed clicking or other noises when opening and closing their mouth. Moreover, many participants reported that, over time, their teeth had become damaged, yellowed, and increasingly sensitive. Table 1 shows the descriptive statistics for the RRS and TEMPS-A on the sample, divided by gender. The results highlight significant differences between men and women: female participants have higher scores in the “Brooding” and “Depression” dimensions of the RRS and in the “Cyclothymic” and “Anxious” factors of the TEMPS-A. Subsequently, the RRS subscales (“Brooding”, “Reflection”, “Depression”) were considered as dependent variables, while the TEMPS-A temperamental factors (“Cyclothymic”, “Depressive”, “Irritable”, “Hyperthymic” and “Anxious”) were included as independent variables in three linear regression models, with the aim of analyzing the possible associations between affective temperamental dimensions and depressive rumination in subjects with bruxism. The results showed that the models accounted for 49.6 %, 24.1 %, and 55.5 % of the total variance for “Brooding” (*F* = 46.56; *df* = 5; *p* ≤ 0.0001), “Reflection” (*F* = 15.05; *df* = 5; *p* ≤ 0.0001), and “Depression” (*F* = 59.16; *df* = 5; *p* ≤ 0.0001), respectively. Moreover, the regression analysis showed that Cyclothymic and Depressive temperaments were predictors of “Brooding” (*β* = 0.361; *p* ≤ 0.0001; *β* = 0.630; *p* ≤ 0.0001) and “Depression” (*β* = 1.072; *p* ≤ 0.0001; *β* = 1.577; *p* ≤ 0.0001), while the Cyclothymic temperament was a predictor of “Reflection” (*β* = 0.402; *p* ≤ 0.0001) (Table 2).

**Table 1.** Descriptive statistics of the total sample and gender differences.

	**Cronbach's *α***	**Total Sample**		**Males (*n* = 57)**		**Females (*n* = 186)**		**Student *t* Test**	

		*Mean*	*S.D.*	*Mean*	*S.D*.	*Mean*	*S.D.*	*t*	*p*
**RRS**									
**Depression**	0.93	26.73	8.90	23.86	9.06	27.61	8.68	−2.822	**0.005**
**Brooding**	0.81	11.43	3.92	9.93	3.76	11.90	3.85	−3.371	**0.001**
**Reflection**	0.77	9.77	3.51	9.02	3.54	10.01	3.47	−1.869	0.063
**TEMPS-A**									
**Cyclothymic**	0.85	5.93	3.52	4.81	3.76	6.27	3.37	−2.782	**0.006**
**Depressive**	0.76	3.23	2.33	2.67	2.47	3.41	2.26	−2.122	0.035
**Irritable**	0.72	1.48	1.70	1.54	1.83	1.46	1.65	0.339	0.735
**Hyperthymic**	0.72	3.93	2.28	4.51	2.27	3.75	2.56	2.212	0.028
**Anxious**	0.67	1.67	1.10	1.16	1.08	1.83	1.07	−4.173	**<0.0001**

**Table 2.** Linear regression analysis.

Dependent variable	Predictors	Unstandardized coefficients		Standardized coefficients	*t*	*p*

		*B*	*S.E*.	*Beta*		
“Brooding” ^a^ (Model 1)	(Constant)	6.747	0.527		12.808	**<0.0001**
	Cyclothymic	0.361	0.065	0.325	5.515	**<0.0001**
	Depressive	0.630	0.102	0.374	6.158	**<0.0001**
	Irritable	0.237	0.129	0.102	1.837	0.067
	Hyperthymic	−0.054	0.084	−0.031	−0.646	0.519
	Anxious	0.219	0.185	−062	1.187	0.236
“Reflection” ^b^ (Model 2)	(Constant)	6.802	0.579		11.747	**<0.0001**
	Cyclothymic	0.402	0.072	0.403	5.580	**<0.0001**
	Depressive	0.266	0.112	0.176	2.360	0.019
	Irritable	−0.117	0.142	−0.056	−0.827	0.409
	Hyperthymic	−0.014	0.092	−0.009	−0.148	0.883
	Anxious	−0.026	0.203	−0.008	−0.128	0.889
“Depression ^c^ (Model 3)	(Constant)	16.947	1.124		15.080	**<0.0001**
	Cyclothymic	1.072	0.140	0.424	7.670	**<0.0001**
	Depressive	1.557	0.218	0.407	7.140	**<0.0001**
	Irritable	0.113	0.257	0.021	0.412	0.681
	Hyperthymic	−0.398	0.179	−0.102	−2.220	0.027
	Anxious	−0.124	0.394	−0.015	−0.315	0.753

Note: ^a^ *R* = 0.704; *F* = 46.57; *p* ≤ 0.0001; ^b^ *R* = 0.491; *F* = 15.05; *p* ≤ 0.0001; ^c^ *R* = 0.491; *F* = 15.052; *p* ≤ 0.0001.

## 4. Discussion

The results of this study demonstrate the strong connection between certain types of affective temperaments and the tendency toward depressive rumination in individuals with bruxism. In particular, it was observed that the depressive and cyclothymic temperaments act as significant predictors of this type of rumination, thus highlighting the importance of stable emotional traits in the onset and maintenance of this parafunctional behavior. These findings align with previous studies that described how occlusion can activate the hypothalamic-pituitary-adrenal (HPA) axis, thus leading to increased physical and emotional stress, which, in turn, heightens muscle activity related to bruxism [22]. One key point of this study is the association between bruxism-related behaviors and the depressive temperament. Previous research has shown that dysfunctional chewing contributes to the persistence or recurrence of depressive episodes over time [23], potentially creating a negative cycle in which emotional distress intensifies, thus reinforcing bruxism. Furthermore, recent studies indicated that individuals with high levels of chewing behaviors tend to experience greater orofacial pain and a reduced ability to manage stress [24], which could explain why these patients exhibit a noticeable decline in their quality of life. Regarding the cyclothymic temperament, its connection to the “Brooding” (negative rumination) and “Reflection” (self-critical reflection) subscales of the RRS suggest that frequent emotional oscillations may hinder the stress regulation and promote ineffective coping strategies. Some authors have proposed that this emotional instability may increase the vulnerability to bruxism by constantly activating the sympathetic nervous system and deregulating the autonomic nervous system, particularly the vagus nerve [9]. Moreover, this difficulty in regulating emotions could also affect the physiological stress response. However, another study showed that the relationship between cyclothymic traits and bruxism may depend on variables such as generalised anxiety or chronic fatigue [25]. Additionally, the identified gender differences found were relevant. The female participants scored higher, and therefore more pathologically, in depressive rumination and affective temperaments such as depressive and anxious traits. This aligns with previous studies which indicated that women tend to use more coping strategies focused on repetitive and negative thinking [26]. However, it has also been reported that men may exhibit a greater severity in the physical signs of bruxism. This suggests the existence of moderating factors such as personal coping styles or physiological responses to stressful situations [27]. These affective temperaments, according to Akiskal [18], represent stable personality dispositions that may constitute vulnerability factors for the development of mood disorders. From this perspective, bruxism cannot be interpreted as a peripheral response to stressful factors, but rather as part of a broader psycho-emotional functioning in which affective and cognitive regulation may play crucial roles. These results highlight the importance of incorporating therapeutic strategies aimed at emotional management and reducing dysfunctional chewing. However, these implications should be considered preliminary and warrant further investigation through controlled studies specifically designed to test their efficacy in bruxism populations. Scientific evidence has linked bruxism as a potential cause of tinnitus [28]. Tinnitus is a debilitating symptom that can have various origins and may also be present in individuals without hearing loss [29]. In summary, the study showed the psychological traits linked to the emotional aspects of bruxism in subjects.

## 5. Limitations and future perspectives

Future research should further explore these findings and assess the impact of combined treatments to optimize the management of bruxism from different perspectives. Our results should be interpreted with caution due to some limitations. The small sample size and the cross-sectional design may have reduced the strength of the findings and limited the ability to understand the relationship between affective temperaments and depressive rumination in people with bruxism. Another limitation concerns the sample, which is characterised by a marked prevalence of the female gender; consequently, the generalizability of the results to the male population is limited. A further limitation is the sole use of self-report measures, which could be subject to social desirability bias or perceptual bias, especially in the emotional and cognitive domains. Integration with objective measures (e.g., electromyographic recordings of bruxism, structured clinical assessments or physiological indices of stress) could provide a more complete picture. Further studies should employ longitudinal designs, gender-balanced samples, and integrate objective measures to validate the results obtained through self-report.

## 6. Conclusions

This study highlights the importance of affective temperaments, particularly the depressive and cyclothymic types, in the onset and persistence of bruxism. These temperaments serve as significant predictors of depressive rumination, which, in turn, contributes to bruxism-related behaviors, thus creating a vicious cycle that exacerbates both the psychological and physical aspects of the disorder. Gender differences also emerged, with women showing higher levels of rumination and affective temperaments associated with bruxism, thus suggesting the need for personalized therapeutic approaches. Nevertheless, it is essential to observe the progression of these factors over time through longitudinal studies in order to improve the effectiveness of interventions. Overall, although these findings provide useful insights into the psychological correlates of bruxism, they should be interpreted with caution as they are exploratory. However, exploratory studies such as this one may offer meaningful contributions to the scientific community by opening new lines of inquiry and supporting the development of more comprehensive theoretical models. Prospectively, further research conducted on larger and differently selected samples may strengthen the robustness of these findings and enhance their practical relevance. Further longitudinal and experimental research is needed to clarify causal pathways and evaluate the therapeutic potential of psychophysiological and psychotherapeutic approaches.

## Use of AI tools declaration

The authors declare they have not used Artificial Intelligence (AI) tools in the creation of this article.
